# Supplementary material for: Splice-Junction-Based Mapping of Alternative Isoforms in the Human Proteome
Source: Cell Rep. Author manuscript; Available in PMC 2020 Jan 15. (PMC6961840; doi:10.1016/j.celrep.2019.11.026)
Supplement: 2 [file NIHMS1546469-supplement-2.zip › DF1/PXD006675/RightAtrium/RightAtrium_8_ART3_IPEPFPLPAPGPVPVPGPK.pdf]

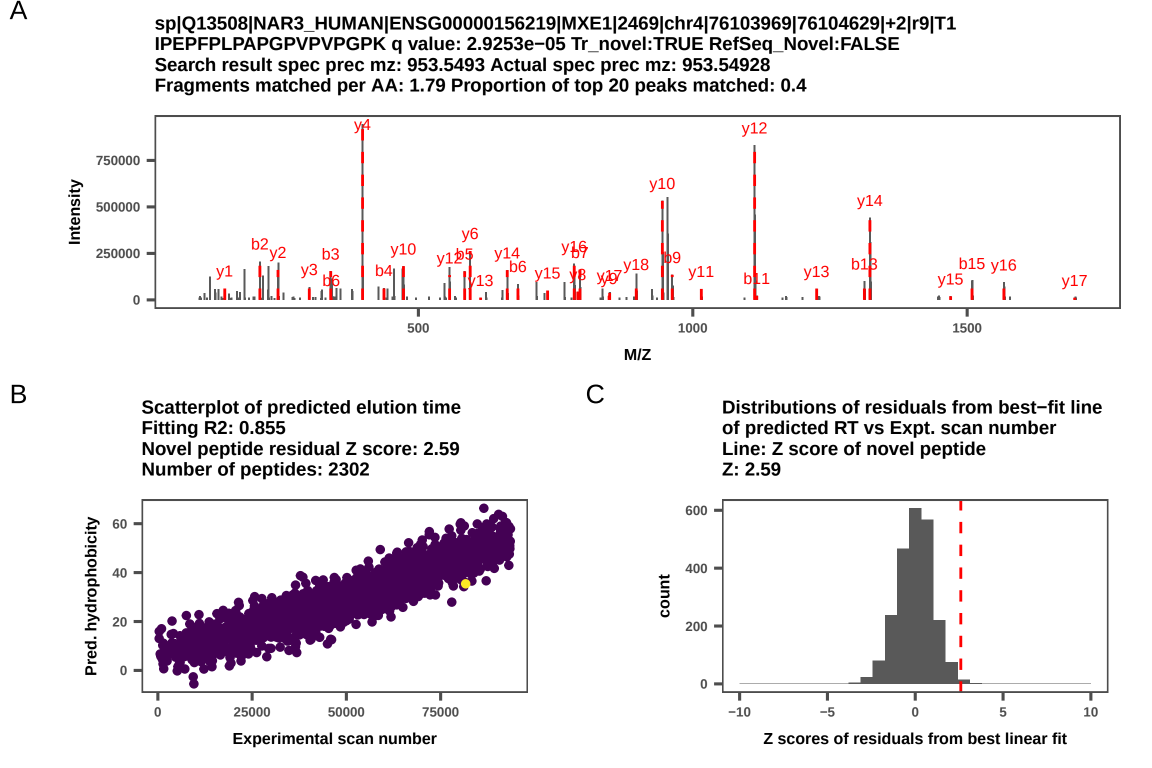
A
sp|Q13508|NAR3_HUMAN|ENSG00000156219|MXE1|2469|chr4|76103969|76104629|+2|r9|T1
IPEPFPLPAPGPVPVPGPK q value: 2.9253e−05 Tr_novel:TRUE RefSeq_Novel:FALSE
Search result spec prec mz: 953.5493 Actual spec prec mz: 953.54928
Fragments matched per AA: 1.79 Proportion of top 20 peaks matched: 0.4
Intensity
750000
500000
250000
0
500
1000
1500
M/Z
y1
b2
y2
y3
b3
b6
y4
b4
y10
y12
b5
y6
y13
y14
b6
y15
y16
b7
y8
y9
y17
y18
y10
b9
y11
y12
b11
y13
b13
y14
y15
b15
y16
y17
B
Scatterplot of predicted elution time
Fitting R2: 0.855
Novel peptide residual Z score: 2.59
Number of peptides: 2302
Pred. hydrophobicity
60
40
20
0
0
25000
50000
75000
Experimental scan number
C
Distributions of residuals from best−fit line
of predicted RT vs Expt. scan number
Line: Z score of novel peptide
Z: 2.59
count
600
400
200
0
−10
−5
0
5
10
Z scores of residuals from best linear fit
